# 晚期肺腺癌靶向耐药后小细胞癌转化2例并文献复习

**DOI:** 10.3779/j.issn.1009-3419.2022.102.41

**Published:** 2022-11-20

**Authors:** 洁琼 吴, 敦强 任, 冰倩 易, 焕焕 毕, 艳梅 邵, 红梅 王

**Affiliations:** 1 266000 青岛，青岛大学附属医院呼吸与危重症医学科 Department of Respiratory and Critical Care Medcine, The Affiliated Hospital of Qingdao University; 2 266000 青岛，青岛大学医学部 School of Medicine, Qingdao University, Qingdao 266000, China

**Keywords:** 小细胞癌转化, *EGFR*基因突变, *RB1*, *TP53*, Small cell carcinoma transformation, *EGFR* gene mutation, *RB1*, *TP53*

## Abstract

具有表皮生长因子受体（epidermal growth factor receptor, *EGFR*）敏感突变的晚期非小细胞肺癌（non-small cell lung cancer, NSCLC）患者应用EGFR酪氨酸激酶抑制剂（EGFR-tyrosine kinase inhibitors, EGFR-TKIs）治疗可取得良好的疾病控制，但不可避免会产生耐药。其中3%-10%左右的耐药机制为小细胞癌转化。本文报道2例IV期肺腺癌存在*EGFR*突变、经EGFR-TKIs治疗后疾病得到控制的病例。病例1发生小细胞癌转化前的无进展生存期（progression-free survival, PFS）为16个月，病例2发生小细胞癌转化前的PFS为24个月。疾病进展后再次活检提示小细胞癌转化。经后续治疗再次稳定，病例1发生小细胞癌转化后的PFS为6个月，总生存期（overall survival, OS）暂未出现，病例2发生小细胞癌转化后的PFS为8个月，OS为36个月，显著延长了患者生存。同时对此类耐药突变进行文献复习。对于晚期NSCLC存在敏感突变的患者，经EGFR-TKIs治疗耐药后进行二次组织病理检测，根据不同耐药机制选择后续治疗对疾病全程管理十分必要。

## Abstract (en)

Treatment of advanced non-small cell lung cancer (NSCLC) patients with epidermal growth factor receptor (*EGFR*) mutation with EGFR-tyrosine kinase inhibitors (EGFR-TKIs) can achieve good disease control, but it will inevitably produce drug resistance. About 3%-10% of the resistance mechanism is small cell transformation. Two cases of stage IV lung adenocarcinoma with *EGFR* mutation were reported and the disease was controlled after EGFR-TKIs treatment. In case 1, progression-free survival (PFS) before small cell carcinoma transformation was 16 months, and in case 2, PFS before small cell carcinoma transformation was 24 months. Subsequent biopsy after disease progression indicated a shift to small cell lung cancer. Case 1 PFS after small cell carcinoma transformation was 6 months, and case 2 PFS after small cell carcinoma transformation was 8 months, and overall survival (OS) was 36 months, which significantly prolonged the patient's survival. At the same time, the literature of such drug resistance mutations was reviewed. For patients with advanced NSCLC with sensitive mutations, it is necessary to conduct secondary histopathological tests after TKIs treatment resistance, and select subsequent treatment according to different resistance mechanisms for the whole course of disease management.

肺癌是发病率和病死率均居高位的恶性肿瘤类型。其中非小细胞肺癌（non-small cell lung cancer, NSCLC），尤其肺腺癌因大多存在基因突变从而可行靶向治疗。基因突变类型包括表皮生长因子受体（epidermal growth factor receptor, *EGFR*）突变（27%）、间变性淋巴瘤激酶（anaplastic lymphoma kinase, *ALK*）重排（3%-7%）、间质-上皮细胞转化因子（mesenchymal-epithelial transition factor, *MET*）扩增（1%-3%）、受体酪氨酸激酶转染时重排（rearranged during transfection, *RET*）融合（1%-2%）、科尔斯顿鼠肉瘤病毒原癌基因（Kirsten rat sarcoma viral oncogene, *KRAS*）突变（20%-25%）等^[1, 2]^。其中*EGFR*基因突变主要与外显子19缺失及外显子21 L858R替换突变相关，称为经典突变类型。G719X、G719A、L861Q、S768I称为非经典突变类型。EGFR酪氨酸激酶抑制剂（EGFR-tyrosine kinase inhibitors, EGFR-TKIs）是具有*EGFR*敏感突变的晚期NSCLC的一线标准治疗方案。患者大多在接受治疗的12个月-18个月后产生获得性耐药。经重复活检证实，其中与发生小细胞肺癌（small cell lung cancer, SCLC）类型转化相关的获得性耐药占3%-10%^[3-6]^。肿瘤病理类型发生转化的机制并不完全清楚，目前最被接受的假设是肺腺癌和SCLC共同的前体细胞，即肺泡II型上皮细胞在TKIs药物的压力选择下，加上抑癌基因视网膜母细胞瘤蛋白1（retinoblastoma protein 1, *RB1*）和肿瘤蛋白P53（tumor protein p53, *TP53*）的缺失，最终转化为SCLC。转化型SCLC的治疗目前尚无明确指南，经既往研究显示，转化型SCLC预后差，转化后治疗的中位无进展生存期（progression-free survival, PFS）仅为6个月，明显低于原发SCLC 8个月-13个月的中位生存期^[7]^。从原发SCLC衍生出的治疗策略在临床上显示出的效果不佳，因此需寻找新的治疗手段。本文通过比较具有*EGFR*经典突变与*EGFR*罕见突变的肺腺癌发生SCLC类型转化后的临床表现及后续治疗，回顾了与转化型SCLC有关的文献，在转化型SCLC发生的机制、临床表现、治疗及预后方面进行讨论。

## 1 病例资料

病例1，73岁，女性，痰血2个月就诊。入院时血清肿瘤标志物癌胚抗原（carcinoembryonic antigen, CEA）、糖类抗原199（carbohydrate antigen 199, CA199）、糖类抗原724（carbohydrate antigen 724, CA724）水平高。胸部增强电子计算机断层扫描（computed tomography, CT）提示左肺下叶占位（46 mm×37 mm），恶性胸腔积液（图 1）。2020年1月6日行第1次肺穿刺活检提示中分化腺癌，甲状腺转录因子-1（thyroid transcription factor-1, TTF-1）（+），天冬氨酸蛋白酶A（novel aspartate peptidase A, Napsin A）（+），程序性死亡配体1（programmed cell death ligand 1, PD-L1）-22C3（-）（图 2）。基因检测仅提示*EGFR*外显子19 Del阳性突变。确诊时分期为T2N2M1a IV期。依据基因检测结果予以患者吉非替尼（易瑞沙）靶向治疗。9个月后患者入院行疗效评估，血清CEA、CA199、CA724水平均较前下降，行肺增强CT提示左肺下叶占位较前缩小（39 mm×31 mm）（图 1）。评价为疾病稳定（stable disease, SD），提示先前靶向治疗有效。因患者临床症状较前明显加重，影像学较入院时缩小，但对比前次评价疾病进展（progressive disease, PD），一代EGFR-TKIs应用近10个月，考虑出现耐药。期望早期发现T790M突变，在肿瘤负荷较小时更换更有效药物，因此于2020年11月2日行第2次肺穿刺活检提示中分化腺癌，PD-L1-22C3[肿瘤细胞阳性比例分数（tumor proportion score, TPS） < 1%]，基因检测提示*EGFR*外显子19 Del突变；*EGFR*外显子20 T790M突变。因出现T790M耐药基因，遂改为奥希替尼（泰瑞沙）继续行靶向治疗。后患者因咳嗽症状加重入院，复查血清肿瘤标志物胃泌素释放肽前体（gastrin-releasing peptide, Pro-GRP）、CEA、神经元特异性烯醇化酶（neuron-specific enolase, NSE）、细胞角蛋白19片段（cytokeratin 21-1, Cyfra21-1）高，肺CT提示左肺下叶占位较前明显增大（77 mm×81 mm）（图 2），评价为PD。行第3次肺穿刺活检提示肺小细胞癌，细胞角蛋白（cytokeratin, CK）（+），TTF-1（+），CD56（灶+），Ki-67阳性率约60%，突触核蛋白（synuclein, Syn）（+）（图 2）。因出现小细胞表型转化，故在奥希替尼靶向治疗的基础上，联合针对SCLC的EP（依托泊苷+卡铂）化疗方案，共6个周期。期间患者出现脑转移，疗效评价为PD，予以脑部放疗21 Gy及安罗替尼治疗，共4个周期。后因患者出现消瘦、无力症状，考虑PD且患者不能耐受肺穿刺活检的风险，遂进行了外周血样本的采集，行高通量基因检测。结果显示*EGFR*外显子19 Del阳性突变、*RB1*突变和*TP53*突变。后患者仅接受支持性治疗。

**图 1:**
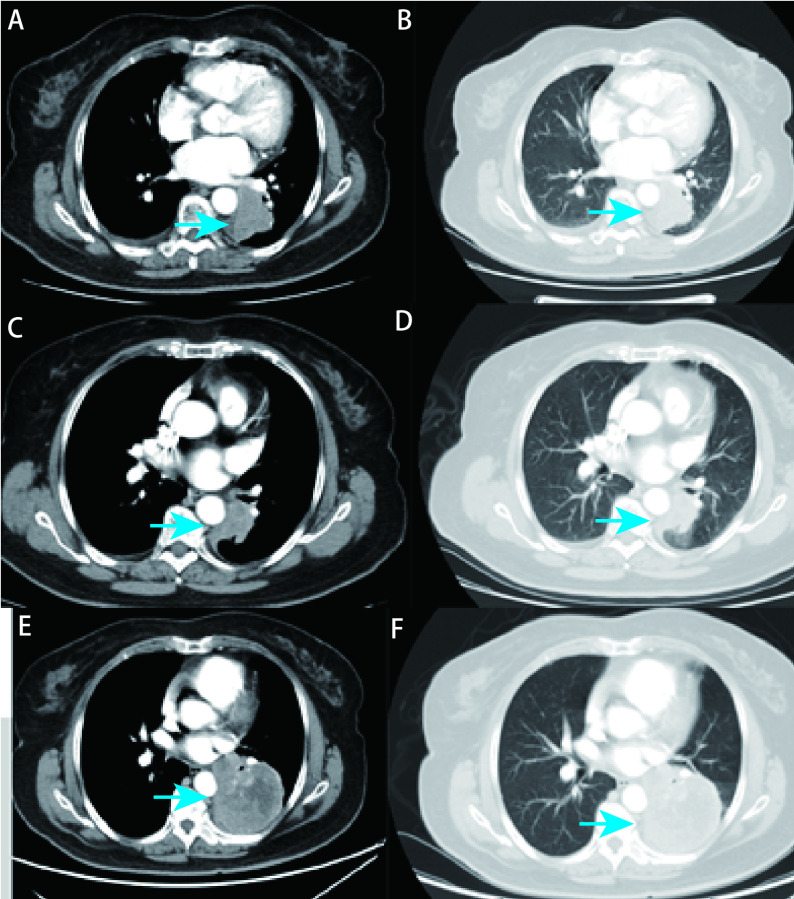
病例1在诊断和治疗中不同时间节点的胸部CT表现。A、B：患者确诊时的胸部CT表现，左肺下叶占位，病灶大小为46 mm×37 mm（A：纵隔窗；B：肺窗）；B：患者确诊时的胸部CT表现，左肺下叶占位，病灶大小为46 mm×37 mm；C、D：靶向治疗9个月后，病灶较前缩小，为39 mm×31 mm（C：纵隔窗；D：肺窗）；E、F：确诊为小细胞肺癌，病灶较前明显增大，77 mm×81 mm（E：纵隔窗；F：肺窗）。 Chest CT findings of case 1 at different time points during diagnosis and treatment. A, B: Chest CT findings of the patient at the time of diagnosis, mass in the lower lobe of the left lung, lesion size of 46 mm×37 mm (A: mediastinum window; B: lung window); C, D: After 9 months of targeted therapy, the lesion was 39 mm×31 mm smaller than before(C: mediastinum window; D: lung window); E: Diagnosed as lung small cell carcinoma, the lesion was significantly enlarged, 77 mm×81 mm (E: mediastinum window; F: lung window). CT: computed tomography.

**图 2:**
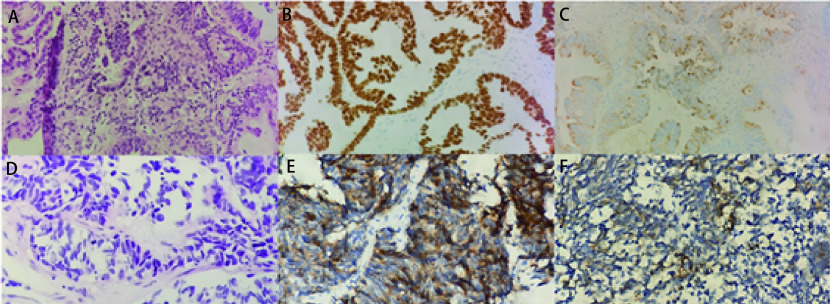
病例1组织活检先后确诊为肺腺癌和小细胞肺癌。A：肺腺癌（HE染色，×40）；B：TTF-1（+）（免疫组化染色，×40）；C：Napsin A（+）（免疫组化染色，×40）；D：小细胞肺癌（HE染色，×40）；E：Syn（+）（免疫组化染色，×40）；F：CD56（+）（免疫组化染色，×40）。 Case 1 was diagnosed as lung adenocarcinoma and small cell lung cancer by biopsy. A: Lung adenocarcinoma (HE staining, ×40); B: TTF-1 (+) (immunohistochemical staining, ×40); C: Napsin A (+) (immunohistochemical staining, ×40); D: Small cell lung cancer (HE staining, ×40); E: Syn (+) (immunohistochemical staining, ×40); F: CD56 (+) (immunohistochemical staining, ×40). TTF-1: thyroid transcription factor-1; Napsin A: aspartate peptidase A; Syn: synuclein.

病例2，60岁，男性，咳嗽、胸闷1个月来诊。吸烟10包年。入院时血清肿瘤标志物CEA、NSE、鳞状上皮细胞抗原（squamous cell antigen, SCC）水平高。肺CT提示右肺上叶占位并右侧纵隔、肺门淋巴结转移（图 3）。伴骨转移、锁骨上淋巴结转移。2018年3月6日行支气管镜检及肺穿刺活检提示浸润性腺癌。免疫组化：TTF-1（+），Napsin A（-），角蛋白7（cytokeratin 7, CK7）（+），角蛋白20（cytokeratin 20, CK20）（-），Ki-67（+）约5%（图 4）。基因检测提示*EGFR*外显子18 Del-G719X突变。分期为T3N3M1 IV期。行1个周期PP方案（培美曲塞800 mg d1+奈达铂100 mg d2）化疗，后予以阿法替尼靶向治疗。6个月后评价为部分缓解（partial response, PR）。后复查血清CEA逐步升高，但影像学表现仍与前相仿。于2019年10月16日行基因检测，但*EGFR* T790M突变为阴性，故继续原方案治疗。半年后患者因头痛再次来诊，复查血清CEA、SCC水平高，肺CT提示右肺上叶占位范围较前增大（图 3），颅脑磁共振成像（magnetic resonance imaging, MRI）提示多发颅内转移瘤，评价为PD。于2020年3月18日行支气管二次活检，提示SCLC，免疫组化：TTF-1（+），Napsin A（-），Syn（+），嗜铬粒蛋白A（chromogranin A, CgA）（-），CD56（+），p40（-），角蛋白5/6（cytokeratin 5/6, CK5/6）（-），CK7（-），Ki-67（+）约90%（图 4）。诊断为转化型SCLC（广泛期），予以患者针对SCLC的4个周期EP化疗（依托泊苷0.1 g d1-d4，卡铂400 mg d2），疗效评价为疾病稳定（stable disease, SD）。后行胸部局部放疗。2020年11月18日复查病情，胸部增强CT提示病灶较前增大（图 3），且伴有双侧肾上腺、脑、骨等多发部位肿瘤转移。评价为PD。出现肾上腺转移后，因患者一般状况差，出现肠梗阻，一直在当地医院对症治疗，未针对肾上腺转移进行治疗。后患者于2021年4月1日去世，至此总生存期（overall survival, OS）为36个月。

**图 3:**
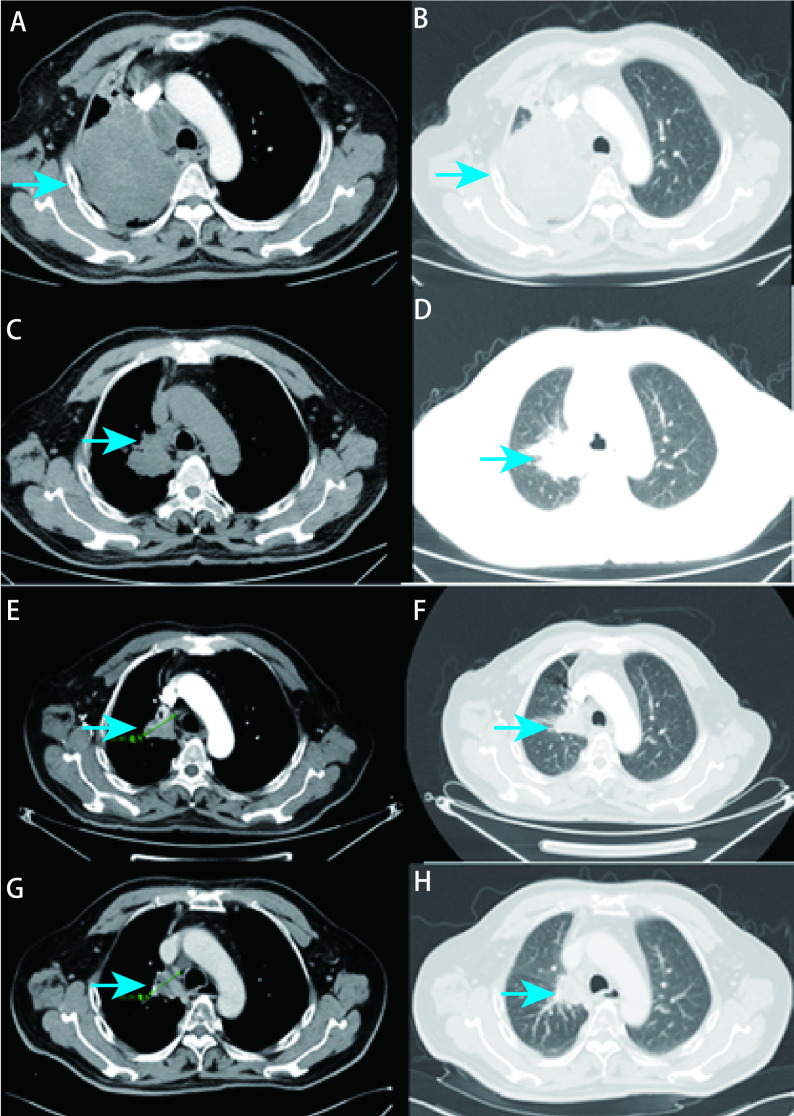
病例2在诊断和治疗中不同时间节点的胸部CT表现。A、B：患者确诊时的胸部CT表现，右肺上叶占位，病灶大小为104 mm×85 mm（A：纵隔窗；B：肺窗）；C、D：靶向治疗后，肺部占位较前缩小，大小为50 mm×40 mm（C：纵隔窗；D：肺窗）；E、F：肺部占位较前增大，出现颅脑转移，评价为PD（E：纵隔窗；F：肺窗）；G、H：确诊为小细胞肺癌，4个周期EP化疗后，肺部占位较前略减小（G：纵隔窗；H：肺窗）。 Case 2 chest CT findings at different time points during diagnosis and treatment. A, B: Chest CT findings of the patient at the time of diagnosis, occupying in the upper lobe of the right lung, lesion size 104 mm×85 mm (A: mediastinum window; B: lung window); C, D: After targeted therapy, the lung space was reduced to 50 mm×40 mm (C: mediastinum window, D: lung window); E: Lung mass increased and brain metastasis appeared, which was evaluated as PD (E: mediastinum window; F: lung window); G: Diagnosed as small cell lung carcinoma, the lung space occupied decreased after 4 cycles of EP chemotherapy (G: mediastinum window; H: lung window). EP: Etoposide+Carboplatin. PD: progressive disease.

**图 4:**
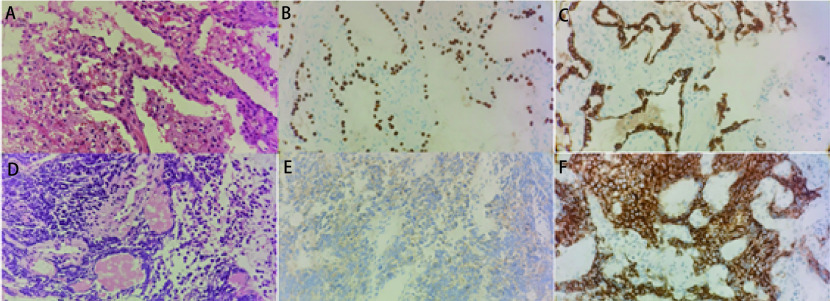
病例2组织活检先后确诊为肺腺癌和小细胞肺癌。A：肺腺癌（HE染色，×40）；B：TTF-1（+）（免疫组化染色，×40）；C：CK-7（+）（免疫组化染色，×40）；D：小细胞肺癌（HE染色，×40）；E：Syn（+）（免疫组化染色，×40）；F：CD56（+）（免疫组化染色，×40）。 Case 2 was diagnosed with lung adenocarcinoma and small cell lung cancer by biopsy. A: Lung adenocarcinoma (HE staining, ×40); B: TTF-1 (+) (immunohistochemical staining, ×40); C: CK-7 (+) (immunohistochemical staining, ×40); D: Small cell lung cancer (HE staining, ×40); E: Syn (+) (immunohistochemical staining, ×40); F: CD56 (+) (Immunohistochemical staining, ×40). CK7: cytokeratin 7.

两组患者的病例特征对比见表 1。

**表 1:** 两组患者的特征对比 Comparison of characteristics between the two groups of cases

Case	Age at diagnosis	Gender	Smoking history	Pathological type of the first biopsy	First-line treatment modalities	Evaluation of best overall response	Pathological type of secondary biopsy	PFS before small-cell transformation	PFS after small cell transformation	OS
Case 1	73	Female	No	LUAD	Gefitinib	SD	SCLC	16 mon	6 mon	Not appear
Case 2	60	Male	Yes	LUAD	Afatinib	PR	SCLC	24 mon	8 mon	36 mon
LUAD: lung adenocarcinoma; SD: stable disease; PR: partial response; PFS: progression-free survival; OS: overall survival; SCLC: small cell lung cancer.										

## 2 讨论

世界第一例SCLC转化病例由Zakowski等^[8]^在2006年报道，一位无吸烟史的*EGFR*外显子19缺失突变的中年女性肺腺癌患者接受EGFR-TKIs治疗后，复发活检时提示出现单纯的SCLC成分，基因检测发现原先的*EGFR*外显子19缺失仍存在。我们在本病例报告中也报道了2例经EGFR-TKIs治疗的*EGFR*突变的晚期肺腺癌患者在发生耐药后二次活检确认发生SCLC转化，发生SCLC转化的时间为16个月-24个月，这与文献报道的肺腺癌初诊到发生SCLC转化的中位时间19个月^[7]^接近。之前的病例报道发现，无吸烟史的具有*EGF*R突变的女性肺腺癌患者发生SCLC转化的概率最高^[9]^，这与我们的病例1情况吻合。与文献报道一致，我们的病例报道也发现，不论是*EGFR*经典突变还是非经典突变，在接受EGFR-TKIs治疗后均可能发生SCLC转化。

目前SCLC转化被认为是化疗、靶向治疗、免疫治疗等产生耐药的机制之一^[10]^。尤其是接受EGFR-TKIs治疗的*EGFR*突变的NSCLC患者，发生SCLC转化的概率最高，这也可能是由于EGFR-TKIs耐药后接受二次活检的患者比例高导致检出率增加所致。关于SCLC转化有两种假设。一种假设认为NSCLC确实可转化为SCLC^[11]^。证据来自于研究证实在原始NSCLC病灶和转化后的SCLC中存在一致的基因表型^[12]^，而*EGFR*突变在SCLC中又是十分罕见的^[12, 13]^。另一种假设认为SCLC与NSCLC在原发肿瘤中同时存在，由于取材的限制，初诊时未检测出SCLC的成分。一线治疗后，NSCLC成分受到抑制后，SCLC成分占主导地位，呈现出了所谓的SCLC转化。而实际上发生SCLC转化的中位时间为19个月，若确在诊断初期即存在SCLC的成分，如此长的治疗时间对EGFR-TKIs药物做出良好反应的同时SCLC缓慢进展，第二种假设难以解释。因此更倾向于第一种假设。

*EGFR*突变型肺腺癌转化为SCLC的机制目前尚未阐明，最被大众接受的假说认为在EGFR-TKIs药物的压力选择下，癌变肺泡II型上皮细胞向SCLC表型转化。更有研究在分子水平上对发生SCLC转化的机制进行解释，转化型SCLC在分子水平上常存在*RB1*和*TP53*突变，导致癌细胞生长、凋亡和DNA修复等的异常，这表明同时存在*RB1*和*TP53*突变很可能与SCLC转化的机制有关^[14-19]^。我们病例1的外周血基因突变检测结果也证实在原来基因突变类型的基础上出现了*RB1*和*TP53*突变，研究^[16, 20, 21]^证实，*EGFR*/*RB1*/*TP53*三者均突变的情况下，使SCLC转化的相对风险增加了43倍。也有学者认为，仅存在*RB1*和*TP53*的突变并不足以引起SCLC转化^[22]^，SCLC转化的发生是多种信号通路异常和分子突变，如PI3K/AKT通路、Notch信号下调、SOX家族突变、*MYC*扩增及AURKA扩增等作用的结果。转化的SCLC与经典的SCLC和NSCLC均不同，转化型SCLC与预后不良有关^[23]^，因此早期的识别十分必要。临床上肺腺癌患者出现PD，同时SCLC相关的指标如NSE、pro-GRP等较前明显升高时，需要考虑出现SCLC转化的可能^[24]^，须及时进行二次活检进行明确。在本文的病例报告中，病例1初始pro-GRP及NSE指标正常，当检测到两者升高时，肺CT提示肿瘤较前增大，二次活检提示发生了SCLC转化，从而及时调整了治疗方案，改善了患者的预后。

转化后的SCLC，目前推荐的一线治疗方案是EP化疗方案，且临床证实有效^[23, 25, 26]^。同时二次活检与原发肿瘤检测出相同的基因组型，所以在SCLC转化后仍沿用靶向药物联合化疗的治疗方式，但很少有研究关注其疗效。本文报道的两例患者中，病例1在发生SCLC转化后即采用了针对SCLC的EP化疗方案联合三代EGFR-TKIs奥希替尼，此选择有效地延长了患者的生存。另外，在一代靶向药物病灶缩小，其后出现缓慢进展时，若能在继续应用一代靶向药物的基础上同时加用局部治疗，如体部立体定向放射治疗（stereotactic body radiotherapy, SBRT）等，使一线治疗药物获益最大化，拖后二线靶向药物使用时间，可能会获得更长生存期；而病例2在发现SCLC转化后，立即停用了靶向药物，仅采用EP方案化疗，此后患者病情快速进展。这提示我们，对于二次活检仅出现SCLC的患者，因穿刺病灶局限性，有未检出其他病理类型的可能性，除了针对SCLC治疗外，应同时兼顾原发肺癌类型的治疗。除了化疗联合EGFR-TKIs外，其他治疗方案也在探索中，有研究^[25]^认为某些靶向表观遗传靶点的药物，如BCL-2抑制剂也可用于基于*EGFR*突变的转化型SCLC的治疗，但疗效尚不明确。免疫疗法也被应用于治疗转化型SCLC的患者，但效果不佳。抗血管生成药物在一定程度上可缓解转化型SCLC的进展^[19, 27]^，而且在经典*EGFR*突变的肺腺癌患者治疗中，靶向联合血管靶向治疗临床有效率高^[28]^，因此或可尝试将其应用于转化型SCLC的病例中。将来通过对SCLC转化前后的肿瘤微环境及细胞亚群进行深入研究，有望发现更多针对转化型SCLC的靶点，从而提高转化型SCLC的疗效。

总之，目前对于转化型SCLC的认识还不足，有关转化型SCLC的研究仅停留在回顾性分析的个案报道层面，期待未来出现更多大型临床试验及前瞻性的研究，明确转化型SCLC的发生机制，从而改善这类患者的预后，提高患者的生存率。对于晚期具有敏感突变的NSCLC患者，当TKIs药物治疗出现耐药后，在患者身体状况及经济条件允许的状况下，应进行二次病理组织活检或液体活检，必要时两者同时进行，避免因组织过小而忽略其他病理成分的存在，然后根据明确的耐药机制进行后续治疗，这对转化型SCLC患者的全程管理十分重要。
